# Expression of P16 in high-risk human papillomavirus related lesions of the uterine cervix in a government hospital, Malaysia

**DOI:** 10.1186/s13000-014-0202-z

**Published:** 2014-11-01

**Authors:** Purushotham Krishnappa, Ibtisam Binti Mohamad, Yip Jo Lin, Ankur Barua

**Affiliations:** Department of Pathology, International Medical University, Kuala Lumpur, Malaysia; Department of Pathology, Hospital Tuanku Ja’afar, Seremban, Malaysia; Department of Community medicine, International Medical University, Kuala Lumpur, Malaysia

**Keywords:** Immunohistochemistry, Human papillomavirus, Cervix, p16INK4A

## Abstract

**Background:**

Cervical cancer is one of the most common cancers affecting women worldwide. It is well established that human papilloma virus (HPV) infection is the prime risk factor in the development of cervical cancer. The current screening and diagnostic tests have limitations in identifying the range of lesions caused by HPV. The current study aims to evaluate the diagnostic value of p16 immunohistochemical (IHC) investigation in high-risk human papillomavirus (HR-HPV) related lesions of the uterine cervix in Hospital Tuanku Jaafar, Seremban, Malaysia.

**Methods:**

A total of 75 cases were selected from the records of Pathology services, Hospital Tuanku Ja’afar, Seremban. The samples were collected in three separate groups (n = 25 per group) as Carcinoma cervix, Carcinoma in situ and Chronic cervicitis. The demographic data of the patients and the representative paraffin blocks were retrieved from Hospital Tuanku Ja’afar, Seremban. The immunohistochemical staining with p16 and HPV 16 L1 were done on all cases. The staining intensity and density were observed and compared among the three groups of cases.

**Results:**

Immunohistochemistry of p16INK4A staining shows nil (0/25) expression in the cervicitis patients, 72% (18/25) in CIN patients and 100% (25/25) in cervical carcinoma. HPV 16 L1 was positive in 100% (25/25) of cervicitis patients, 96% (24/25) of CIN patients and 40% (10/25) of cervical cancers patients. A chi square test was used to analyze the result and the obtained p value was <0.05.

**Conclusion:**

p16 expression was strongly observed in cervical cancer and minimally observed in cervicitis. Thus indicating p16 immunohistochemistry investigations can aid in diagnosing the different categories of cervical lesions into benign, insitu and malignant.

**Virtual Slides:**

The virtual slide(s) for this article can be found here: http://www.diagnosticpathology.diagnomx.eu/vs/13000_2014_202

## Background

Cervical cancer is the third most commonly diagnosed cancer and the fourth leading cause of cancer death in females worldwide, accounting for 9% (529,800) of the total new cancer cases [1]. Despite the implementation of Pap test that has successfully brought dramatic reduction in the incidence and mortality worldwide caused by cervical cancer from 50% to 75% [2], there are still a substantial amount cervical cancers occurring in in women who are adequately screened [3], proving diagnostic limitation of the Pap test. It is also estimated that false negative rate for the Pap test for cervical premalignant lesions and cervical cancer lies between 15% to 50% [4] and the false positive rate of approximately 30% [4].

Conversely, conditions such as reserve cell hyperplasia, inflammatory atypia and atypia squamous metaplasia often give rise to significant amount of false positive test result of the Pap test. This leads the patients to repeat the test, or look into unnecessary and more invasive diagnostic procedure to reconfirm the result of the initial positive Pap test and also possibility of overtreatment. Due to the limitation of the current screening programs, demand for a more sensitive and specific test as well as high positive predictive value (PPV) and also negative predictive value (NPV) to improve cervical cancer screening programs and especially to accurately diagnose precancerous lesions are increasing [5,6]. Therefore, it is proposed that histology assessment plays an important role in diagnosing precancerous lesions and also invasive squamous cell carcinoma of the cervix (SCC) [7], but there could be intra-observer and inter-observer diagnostic discrepancies even among panel of pathologist reviewing the slides [8]. Hence to further improve the accuracy of screening test, various IHC biomarkers have been evaluated for its sensitivity and specificity of staining towards precancerous lesions and uterine cervical carcinoma in histological biopsy.

Epidemiological and molecular studies have shown that human papilloma virus (HPV) is the most important etiological agent for cervical carcinogenesis. In cervical lesions, overexpression of p16 is observed and it is thought to be resulted from the increasing level of E2F transcription factor which is releases from pRB after binding to HPV E7 oncoprotein [9].

The association of p16 and HR-HPV in Asia and in more particular Malaysia are still relatively unexplored. This study aims to evaluate the diagnostic value of p16 IHC in differentiating the different categories of HR HPV infected cervical lesions [10].

## Methods

### Research sample

The biopsies referred for histological evaluation of Cervical lesions were studied retrospectively from January 2012 to December 2012, at department of pathology, Hospital Tuanku Ja’afar, Seremban. The study was approved by the International Medical University joint committee for ethics and research. A total of 75 cases were selected from the records of Pathology services, Hospital Tuanku Ja’afar, Seremban. The samples (n = 25 per group) were collected as the following groups:

- Group 1 – Twenty five (25) cases of Carcinoma cervix
- Group 2 - Twenty five (25) cases of Carcinoma in situ
- Group 3 – Twenty five (25) cases of Chronic cervicitis

After obtaining the consent from the relevant authorities of Hospital Tuanku Ja’afar, Seremban, a representative paraffin block of each case along with the patient demographic data were retrieved from Department of Pathology, Hospital Tuanku Ja’afar, Seremban.

All the 75 cases were submitted for IHC with p16 antibody {Mouse monoclonal [2S9A12] to CDKN2A/p16INK4a, Abcam ab 54210} and HPV 16 L1 antibody {Mouse monoclonal [CamVir 1] to HPV 16 L1, Abcam ab 69}.

The IHC methods done in this study followed a standard protocol by the manufacturers with needed optimization. Two pathologists screened the IHC slides independently. A positive and a negative control were run with every batch of IHC staining done. A positive reaction was considered if staining of cells of interest for the antibody in question is observed. The Allred scoring system [11] for IHC staining of the cervical tissue was implemented to allow qualitative and semi-quantitative analysis on the IHC stained slides. The two parameters of interest were staining intensity (SI) and staining density (SD).

**Staining intensity (SI)** was scored according to the following scale:

no visible staining = 0, weak staining = 1+, moderate staining = 2+, and intense staining = 3 + .

**Staining density (SD)** for each antibody was semi-quantified into five main categories based on the percentage of cells being stained positive: 0 = no cells stained positive, 1 = <10% of the cells stained positive, 2 = 10-50% stained positive, 3 = 50-90% stained positive and 4= > 90% of the cells stained positive. The respective grades given to both SI and SD were tabulated and averaged.

## Results

### Age and racial prevalence

Ages of the patients from the 3 groups of cases (cervicitis, CIN, and cervical cancer) ranged from 21 years to 85 years with a mean of age 49.2 years. Patient with cervical cancer (mean age of 58.9 years) was older compared to CIN patients (mean age of 43.4 years). Cervicitis patients were relatively younger with the mean age of 39.7 years.

**Figure 1:**
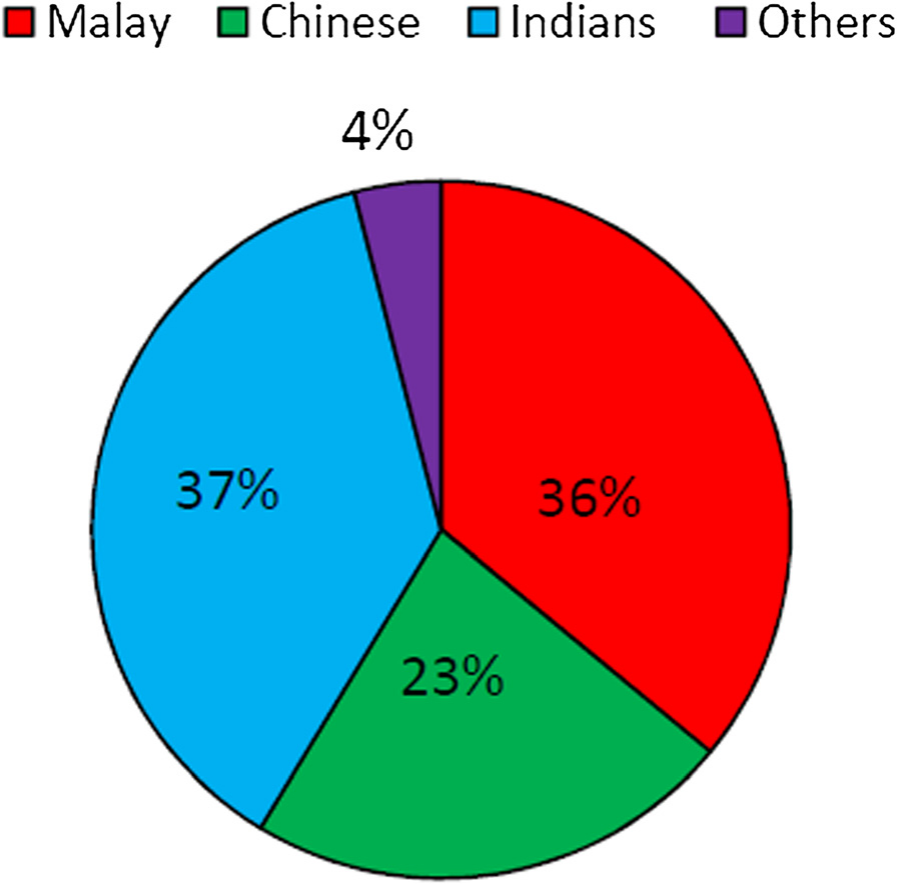
**Showing overall racial prevalence of the sample cohort.**

Overall, Indians stands the largest proportion of 37% (28/75 cases) of having cervical lesions followed very closely by the Malays 36% (27/75 cases) and Chinese 23% (17/75 cases). 4% of the patients are of race other than the 3 main races belonging to Malaysia as shown in the Figure 1.

In the cervicitis group, the Indians (13 cases, 52%) were more commonly affected followed closely by the Malays (8 cases, 32%), and the Chinese (4 cases, 16%). As for the carcinoma-in-situ group of patients, more Malays (10 cases, 40%) were affected than the other races. In the cervical cancer group Malays (9 cases, 36%) were more commonly affected.

**Figure 2:**
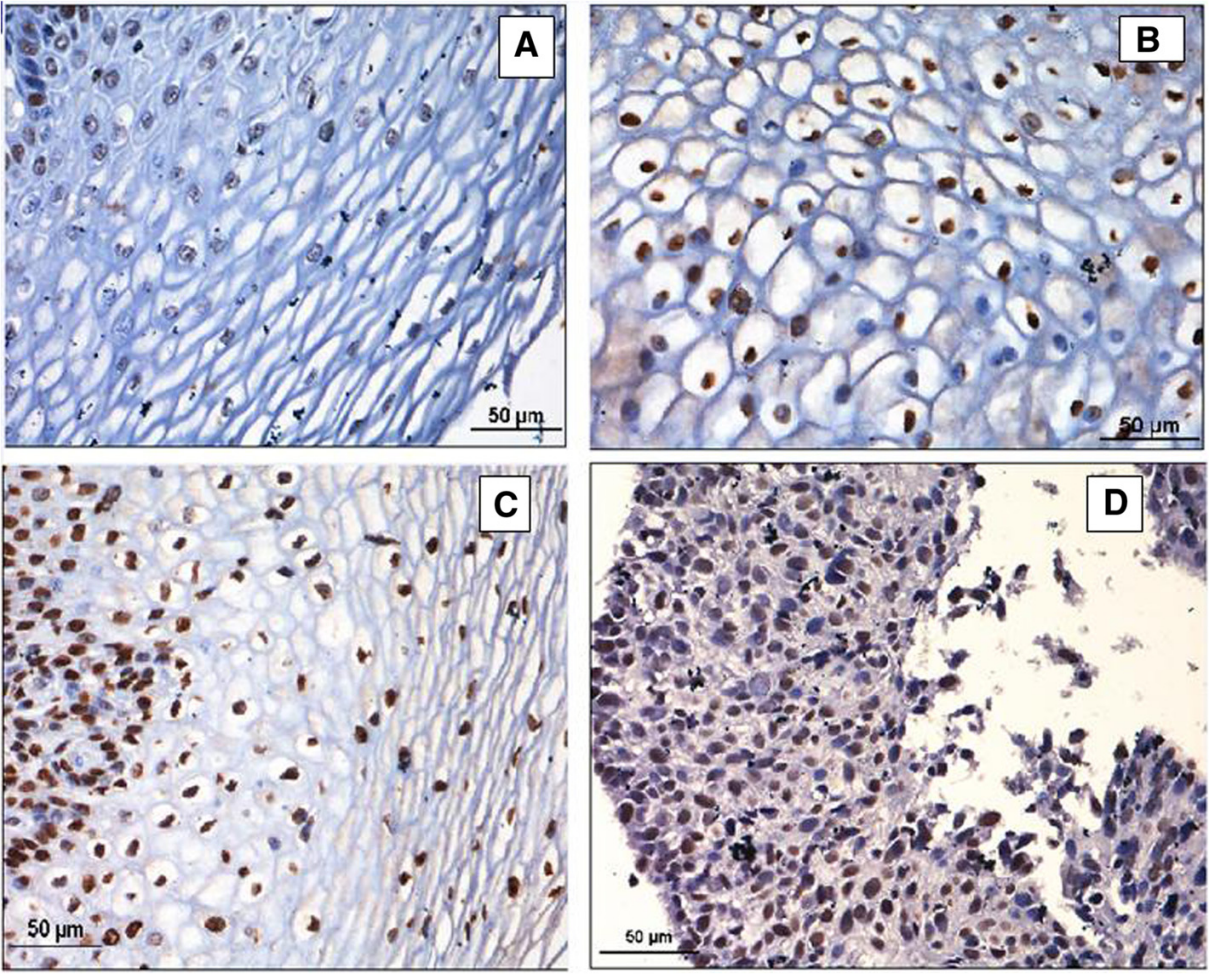
**Showing immunohistochemical staining intensity scores of p16. A-Score 0, B-Score 1, C-Score 2, D-Score 4.**

**Figure 3:**
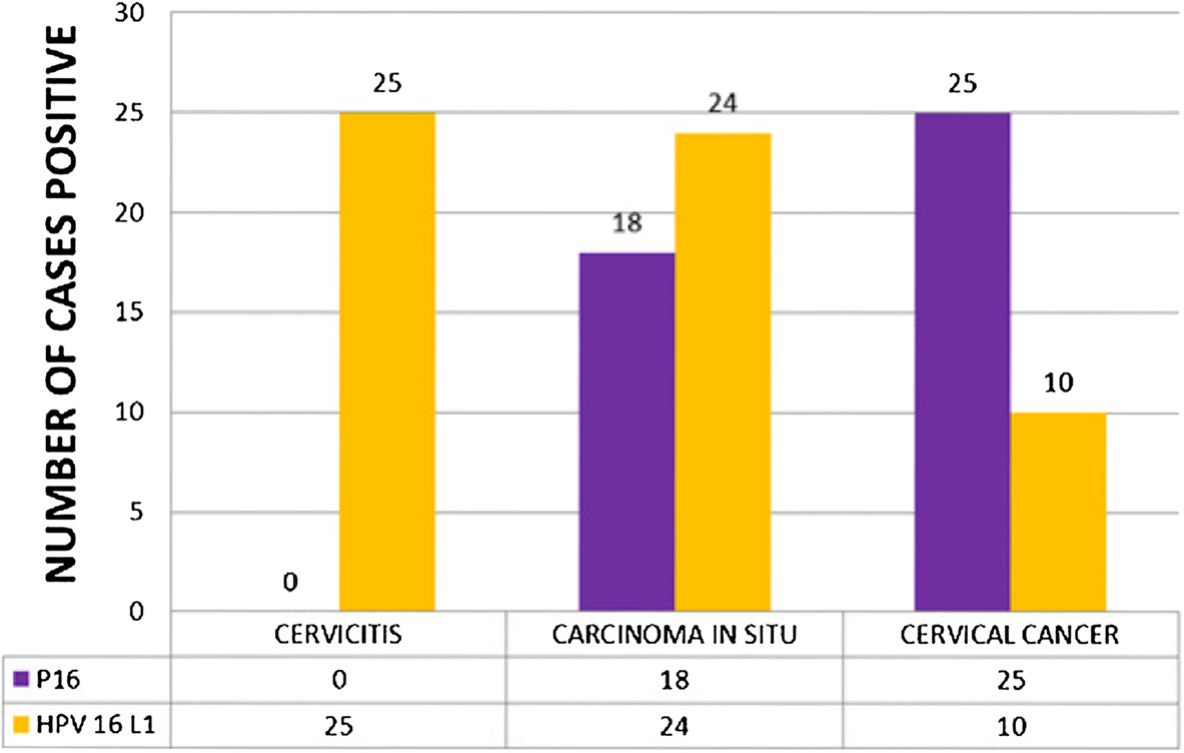
**Showing p16 and HPV 16 L1 positivity.**

**Immunohistochemical staining** for p16 and HPV 16 L1 was done for all 75 cases (100%). Figure 2 shows the p16 IHC staining intensity scores of 0, 1, 2 & 3 labelled as A, B, C & D respectively. Of the 25 cases in the cervicitis group, none of the cases showed p16 expression. Whereas 72% (18/25 cases) of the CIN cases showed p16 positivity and all the cervical cancer cases (100%) showed positivity as shown in the Figure 3. This means that, p16 expression is seen more in cancer group in comparison to in-situ and cervicitis group.

HPV 16 L1 immunohistochemical staining was found positive in all the cervicitis cases ( 100.0%), 24 cases (96.0%) of carcinoma in situ and 10 cases (40.0%) of cervical cancer as shown in the Figure 3.

The Allred scoring system for IHC staining was implemented and the mean intensity and mean density score for each IHC marker were calculated and tabulate in the Table 1. It was observed that HPV 16 L1 had the higher mean intensity score with a score of 1.75 and followed by the p16 with a score of 1.07. Besides that, HPV 16 L1 also had the higher mean density score of 3.16 compared to the p16 with a mean density score of only 1.76.

**Table 1:** **Showing mean intensity and density scores of the p16 & HPPV 16 IHC**

**Antigen (type)**	**No. of cases positive (%)**	**Mean intensity score**	**Mean density score**
p16	43( 57.3)	1.07	1.76
HPV 16 L1	64 (85.3)	1.75	3.16

As the disease status progress from the cervicitis towards the cervical cancer group, it can be seen that the mean intensity score increases from 0.00 (cervicitis), 1.04 (CIN) and lastly to 2.16 (cervical cancer).

Meanwhile, the p16 mean density score also followed the pattern of increase as p16 intensity. The mean density of cell stained increases from 0.4 (cervicitis), 1.6 (CIN) and lastly 3.68 (cervical cancer).

On the other hand, the HPV 16 L1 mean staining intensity showed inversed relationship as compared to those seen in p16 marker. The mean score decreases from 2.32 (cervicitis), CIN (2.12) and 0.84 for cervical carcinoma. The HPV 16 L1 mean density score also observed the same decrease from 3.68 (CIN) to cervical carcinoma (1.8).

The tabulated p16 expression and the disease categories was ran onto statistical test using SPSS version 18 and the chi square test was calculated and the p value was <0.05.

## Discussion

P16INK4A is a gene that is expressed by host cells in response to HPV infection, and is not normally expressed in non-transformed cells [12]. The p16 gene is on chromosome 9p21-22, and maps for a cyclin dependent kinase (cdk)-4 inhibitor. It normally decelerates the cell cycle at the G1-S phase checkpoint by inhibiting the cdk that phosphorylates and inactivates pRb. It is specifically binds to cyclin D-cdk4/6 complex, and thereby prevents the conformational change activated by phosphorylation of pRb that will release transcription factor E2F from the E2F/pRb complex, and so causes a halt at the checkpoint before entering into the S1 phase [13].

Upon functional inactivation of pRb by E7 oncogene, there is increased activity of cyclin dependent kinases that propel the cell into the S phase of cell cycle. As p16 expression is involved in a negative feedback or a down regulator of cell proliferation but this inhibitory function is proved to be ineffective in the context of high risk HPV integrated infection due to the substantially high amount of E6 and E7 oncogene present in the infected host cells. Therefore it is a sensitive surrogate marker for such HPV infections, specifically for integration of viral E6 and E7 into squamous epithelial cell genome.

Only the high risk HPV subtypes have the ability to integrate into the replicating basal and parabasal epithelial stem cell genome resulting in overexpression of p16INK4A protein from the basal layers up, and perhaps reflecting malignant transformation. As a result, the p16INK4A staining pattern for the high risk HPV subtypes is those of strong, diffuse staining of both nuclei and cytoplasm [14]. In contrasts, the immunostaining pattern associated with episomal infection by low risk HPV subtypes displayed weak, focal staining of nuclei and cytoplasm in the intermediate and superficial layers only.

In addition, p16INK4A is not only expressed in cervical tissue but also in normal tissues. In adults, p16INK4A is found in normal proliferative endometrium, oesophageal squamous epithelium, breast ductal epithelium, antral gastric glands, salivary glands, Langerhans cells in pancreas, anterior pituitary, Sertoli and Leydig cells in testis. Whereas in infants, p16INK4A expression is limited to thymic corpuscles and only rarely in pancreatic epithelial cells [15].

The other Biomarkers have been used to evaluate the cervical neoplastic lesions. Yanxia et al showed the down regulation of miR-143 was a promoting factor for the occurrence of cervical SCC, and related to the infection of HPV16. The down regulation of miR-143 was also related with tumor size and lymph node metastasis [16].

Hongxiu et al showed that D2-40 may be a helpful marker for distinguishing CIN1 from CIN2/3 in pathological practice [17]. Hongqian et al showed the Potential clinical usefulness of the hTERC FISH in distinguishing patients with clinically significant cervical lesions from those that are insignificant lesions, especially in HPV-infection patients [18].

Based on studies published so far, p16 could be potentially be utilized in the detection of HR-HPV in Pap smears. Many reports have described the successful application of p16 immunohistochemistry (IHC) to liquid based and conventional Pap smears, with good concordance between p16 and Pap smear results [19]. Ekalaksanan et al [20], stated that all cases of HSIL and squamous carcinoma in his research showed p16 positive by IHC. Similar results were obtained by Klaes et al [12]. Some studies have also suggested the utility of p16 IHC in the interpretation and triage of Pap smears demonstrating ASCUS and low-grade squamous intraepithelial lesions (LSIL) [21]. Cervical intraepithelial neoplasia (CIN1) with diffuse p16 staining has a greater risk of progression to HSIL over p16 negative cases [22]. A possible reason for the lower expression of p16INK4A in low grade lesions may be because a certain percentage is thought to be caused by low risk HPV types. This is because the affinity of the E7 protein of low risk HPV for pRb is much lower than that of high risk HPV types, there would not be any overexpression of p16INK4A [23].

The study done by Iana et al concluded that in large numbers of sections we were able to prove that immunohistochemical detection p16^INK4a^ expression can be used as a specific diagnostic marker of all degrees of cervical dysplasia and cervical cancer, and possibly as a surrogate marker for HPV infection, due to the relationship between p16^INK4A^ and HPV E7 inactivated RB protein [24].

Benvolo et al has stated that, in this context, p16 testing can substantially improve the conventional morphological diagnosis of cervical preneoplastic lesions [25]. Nevertheless only a few literatures have analyzed the correlation between p16 overexpression and HPV infection [25]. However, morphological criteria alone are not sufficient to distinguish lesions that may regress from those that might progress and persists. Therefore, the evaluation of HR-HPV infection as well as p16 immunoreactivity, could be useful tools of particular clinical value in identifying cases with a higher possibility to progress to high grade lesions. In a study done by Maria et al study, they managed to show a statistically significant inter-relationship occurring between p16 overexpression and HR-HPV infection with a 84% sensitivity, 98% specificity, 97% PPV, 86% NPV [25].

All these literatures proved that p16 seems likely to be superior to other methods of HR-HPV detection because it can assess gene expression and not merely due to the presence of virus. Therefore, p16INK4A immunostaining may play a useful role in classifying the different groups of lesions.

## Conclusion

The study showed a significant statistical difference in the expression of p16 between the low grade and high grade cervix lesions. P16 immunohistochemistry can be used as a useful diagnostic tool for detecting high grade cervical lesions.
